# Guidelines for rating Global Assessment of Functioning (GAF)

**DOI:** 10.1186/1744-859X-10-2

**Published:** 2011-01-20

**Authors:** IH Monrad Aas

**Affiliations:** 1 Department of Research, Vestfold Mental Health Care Trust, Tönsberg, Norway

## Abstract

**Background:**

Global Assessment of Functioning (GAF) is a scoring system for the severity of illness in psychiatry. It is used clinically in many countries, as well as in research, but studies have shown several problems with GAF, for example concerning its validity and reliability. Guidelines for rating are important. The present study aimed to identify the current status of guidelines for rating GAF, and relevant factors and gaps in knowledge for the development of improved guidelines.

**Methods:**

A thorough literature search was conducted.

**Results:**

Few studies of existing guidelines have been conducted; existing guidelines are short; and rating has a subjective element. Seven main categories were identified as being important in relation to further development of guidelines: (1) general points about guidelines for rating GAF; (2) introduction to guidelines, with ground rules; (3) starting scoring at the top, middle or bottom level of the scale; (4) scoring for different time periods and of different values (highest, lowest or average); (5) the finer grading of the scale; (6) different guidelines for different conditions; and (7) different languages and cultures. Little information is available about how rules for rating are understood by different raters: the final score may be affected by whether the rater starts at the top, middle or bottom of the scale; there is little data on which value/combination of GAF values to record; guidelines for scoring within 10-point intervals are limited; there is little empirical information concerning the suitability of existing guidelines for different conditions and patient characteristics; and little is known about the effects of translation into different languages or of different cultural understanding.

**Conclusions:**

Few studies have dealt specifically with guidelines for rating GAF. Current guidelines for rating GAF are not comprehensive, and relevant points for new guidelines are presented. Theoretical and empirical studies, and international expert panels would be valuable, as well as production of a manual with more information about scoring. Computerised assessment may well be the future.

## Background

Reliable assessment of the problems patients face is important. With regard to the assessment instruments, guidelines for their use are also important [1-5]. Work has been carried out internationally to develop guidelines for psychological tests [6-8], but it is considered that a gap exists between existing standards and the need for regulation of the assessment process. Standardised scoring procedures are important, as they can reduce unintended bias [9-11]. There are many assessment procedures available in psychiatry, but little work has been done with guidelines for these methods [8].

In psychiatry, the severity of illness can be scored by Global Assessment of Functioning (GAF). GAF is known worldwide and it is Axis V of the internationally accepted Diagnostic and Statistical Manual of Mental Disorders, Fourth Edition Text Revision (DSM-IV-TR) [12]. The GAF instrument was analysed in a previous study [13], but questions have been raised as to whether clinician's rate GAF appropriately [14]. GAF is intended to be a generic rather than a diagnosis-specific scoring system. It is constructed as an overall (global) measure of how patients are doing and rates psychological, social, and occupational functioning, covering the range from positive mental health to severe psychopathology. Internationally, GAF recorded values can be either a single score (only the most severe of the symptom and functioning values is recorded) or separate scores for symptoms (GAF-S) and functioning (GAF-F). For both the GAF-S and GAF-F scales, there are 100 scoring possibilities (1-100).

An advantage of GAF is its simplicity [13], but problems have been found with its reliability and validity. Reliability studies show the extreme 20% of raters account for more than 50% of the spread of scores, and deviations can be 20 points or more [15,16]. Overall reliability can be good, but is not sufficient in the routine clinical setting [16-21] and is too low for assessment of change for the individual patient [20]. Concurrent validity [17,18,22-34] and predictive validity [19,23,25,27,35-37] are problematic. There are few empirical results for GAF sensitivity [13].

In general, psychiatric evaluation is too dependent on subjectivity, as assessors may rate psychiatric impairments according to their own experience and attitudes [3]. Rating GAF is no exception to this element of subjective judgement [13]; there is evidence that different professions assign different scores [38,39] and that the scores can be influenced by disagreement on criteria for rating [16], lack of training [22], or problems related to the intrinsic properties of GAF itself [13]. It has also been reported that site of investigation can explain some of the variability [34].

In the present study, guidelines are defined as written instructions that give guidance or recommendations for scoring and consist of some steps that are accepted by clinicians and the scientific community.

Guidelines are important for quality assurance of the assessment [40], and research has demonstrated that variation in guidelines influences the responses given by patients [41]. It should, therefore, be possible to develop better instructions for scoring of GAF [42].

The aims of the present study were to identify the current status of guidelines for rating GAF, points that are relevant for new guidelines, and gaps in knowledge that are of interest for the development of improved guidelines. Gaps in knowledge are defined as points concerning guidelines for scoring GAF where no, or little, research has been done and where it is likely that further development would play a role for improved scoring.

## Methods

A literature review [43-47] was carried out. This was conducted by both hand searching and a search of bibliographic databases in several steps, where steps (a) and (b) represent the necessary 'end of the thread' to start the literature search: (a) from previous work [13], the author had access to literature about relevant issues, namely literature about GAF and other scoring systems, which also includes information about methodology; (b) browsing through journals, which has been recommended as a useful first step before computer searching [44], where each issue of a set of journals for the period January 2000 to December 2009 was searched (*Acta Psychiatrica Scandinavica*, *American Journal of Psychiatry*, *Applied Psychological Measurement*, *Archives of General Psychiatry*, *BMC Psychiatry*, *British Journal of Psychiatry*, *Comprehensive Psychiatry*, *European Journal of Psychological Assessment*, *European Psychiatry*, *Evidence-Based Mental Health*, *International Journal of Testing*, *Journal of Psychiatric Research*, *Psychiatric Bulletin*, *Psychiatric Services*, *Social Psychiatry and Psychiatric Epidemiology*, and *Journal of Clinical Psychiatry*); (c) thorough hand searching: after identification of publications by steps (a) and (b), their reference lists were hand searched for more literature and, by reading total publications, a search for citations to other studies was also conducted.

Each time a relevant publication was identified, the same search for new literature was performed. After several rounds of such hand searching, new relevant references became difficult to find and the search proceeded to steps (d) to (i): (d) search in PubMed, which used experiences from research on search strategies [48,49]. A search was carried out for English language articles from the period January 1990 to December 2009. Search terms were: 'Global Assessment of Functioning OR GAF AND' combined with nine search terms ('guidelines', 'standard', 'reliability', 'validity', 'sensitivity', 'literature review', 'systematic review', 'psychometrics', 'methodology') in nine separate searches. A total of 1,694 studies were identified by this method; (e) Possible missing publications remaining after steps (a) to (d) were controlled for by an Advanced Search in Google Scholar (for both books and articles) for the period from January 1990 to the day the search was performed (22 April 2010). The search terms 'Global Assessment of Functioning psychiatry' (used in 1 common search) identified 17,300 items (mostly publications), and the first 1,000 were screened for relevance. Google Scholar gives information about the number of links to each publication (this is effectively a citation tracking with the most frequently cited publications listed first). The Google Scholar search did not identify any studies that had not been already identified by steps (a) to (d); (f) A search in PsycINFO: this used experiences from research on search strategies [48,49]. A search was carried out for English language articles from the period January 1990 to 28 April 2010. Search terms were: 'Global Assessment of Functioning OR GAF AND' combined with seven search terms ('guidelines', 'instructions', 'standard', 'norm', 'process AND rating', 'process AND scoring', 'methodology') in seven separate searches. A total of 69 studies were identified by this search; (g) A search in The Campbell Collaboration Library of Systematic Reviews was carried out on 22 April 2010. The all-text searches were not limited to a specific time period. Five separate searches were performed (search terms: 'GAF', 'Global Assessment of Functioning', 'psychiatry systematic review', 'psychiatry literature review', 'psychiatry review'). However, this search identified no relevant studies; (h) The abstracts from steps (d) to (f) were screened, with the purpose of identifying literature concerning guidelines for GAF. When this screening started, the researcher was experienced from reading literature from steps (a) to (c). Abstracts were evaluated for inclusion by looking for information on the following issues in relation to GAF: guidelines, instructions, process of rating, methodology, psychometrics (studies with information on validity and reliability), history of GAF, and modifications/changes made. When the screening of abstracts was finished, selected publications were read in their entirety, but it became clear that most of the relevant literature had already been identified by steps (a) to (c); (i) For the selected publications from step (h), the reference lists were hand searched for more literature. New publications that were relevant for inclusion were difficult to find, and the literature search was complete.

The final two steps were as follows: (j) the contribution of each selected publication to the knowledge base for the present study was summarised [44]. Emphasis was placed on points that were relevant for new guidelines and analysis was performed to identify gaps in knowledge; (k) The final set of selected publications is the reference list of the present study. Included publications are original research papers, books, articles and book reviews.

## Results

The literature review identified seven main categories, with a number of points (covered individually below) considered important in relation to further development of guidelines: (1) general points about guidelines for rating GAF; (2) introduction to guidelines, with ground rules; (3) starting at the top, middle or bottom level of the scale; (4) scoring for different time periods and of different values (highest, lowest or average); (5) the finer grading of the scale; (6) different guidelines for different conditions; and (7) different languages and cultures.

Where the presentation of problems concerning guidelines does not require any distinction between the single-scale and dual-scale GAF, no remarks are made about this. Guidelines for scoring single-scale and dual-scale GAF can be quite similar. When the single scale is used, 'whichever is the worse' of the symptom and functioning values is the single value recorded (according to the manual for DSM-IV-TR) [12].

### (1) General points about guidelines for rating GAF

Brief guidelines for rating GAF exist, but their lack of depth is likely to result in subjectivity in rating [5]. They are also different in several respects. An early version of GAF (the Global Assessment Scale (GAS)) had scoring instructions [50], but the publication of DSM-IV-TR updated GAF, with significant changes in these rating instructions [12,27]. The Veterans Administration in the US [5,22] and Norwegian psychiatry services [51] have guidelines. Other systems based on GAF also have guidelines, for example the Modified GAF [24] and Kennedy Axis V [52].

In practice, experienced clinicians operate by forming initial hypotheses and testing them through assessment [53], but they can be faced with dilemmas about which GAF value to choose. If guidelines are going to be of value for rating, they need to be clear, specific and complete. The process of scoring must take account of all the specific properties of GAF [13]. Work with guidelines for psychological tests could form the learning base for further work with guidelines for GAF; for example, the International Test Commission has developed guidelines for using psychological tests [6,7,54,55] and several of the points in these guidelines apply to assessments used in psychiatry.

When assessment instruments are developed, study of the assessment process should be a standard procedure [9], but there has been little interest in guidelines for GAF scoring. International panels of experts have played a limited role in guideline development, and few have compared the content of existing guidelines or investigated what the correct norm for the scoring process should be [3,14,39]. There is limited empirical research on the actual process of scoring, and one study has shown that the actual process agrees well with the concept of GAF [14]; however, the actual process is not necessarily the same as the prescribed process [14]. Before training, practitioners will often choose an incorrect strategy for scoring GAF [22]; for example, they may use the average of the functioning and symptom scores (for the single-scale GAF, only one value is recorded), the least severe of symptoms, or the highest area of functioning [22].

#### Gap in knowledge

In the historical development of GAF, there has been little research on existing guidelines. Few studies have compared the effect of using different existing guidelines for rating and the effect of systematically varying guidelines. We do not know which norms for the guideline are best or whether changed and extended guidelines would improve rating.

### (2) Introduction to guidelines, with ground rules

The introduction to guidelines should give raters a basic understanding of the guidelines' other specifications and what to look for when scoring GAF. However, existing guidelines for rating GAF have different introductions [5,12,50,51]. When different introductions lead raters thinking in different directions, an effect on GAF scores is likely. Developing a good concise introduction should not be considered an irrelevant detail; if it is weak and poorly defined there is a risk that raters will use their individual perspectives to make judgements and use norms from other sources; for example, a clinician working mainly with severely ill patients may unintentionally use this experience as a norm for the less severely ill [5]. However, this has been given little attention in international publications.

The introductory paragraph in a guideline for rating GAF could start by explaining the purpose of rating GAF, for example to score the overall level of functioning or severity of illness [50] and why GAF values are important. Then, a key purpose for the guideline should be given, for example to enhance assessment by describing competent instrument use, to help in standardising rating so that influence of change in the assessor is minimised, and to help in assigning more accurate scores [6,7,56].

In the second paragraph, a definition of what GAF is can be given [13] and an image of the scale(s) provided (with anchor points, key words and examples). The next point could be ground rules for the rating itself. As GAF means rating functioning and symptoms, these terms should be defined, with examples of symptoms and functioning that should and should not be taken into consideration. When rating, all the available information that is important for GAF-S and GAF-F should be considered [14,29], but this information should then be sufficient for good overall judgement of both symptoms and functioning. In both the DSM-IV-TR and the Norwegian instructions, there is a ground rule: 'consider psychological, social, and occupational functioning on a hypothetical continuum of mental health-illness' [12,51,57], but there is little published analysis of how this ground rule is understood by different assessors and how well it works in practice. According to the Norwegian guidelines, this ground rule means that symptoms (and functioning) should be viewed in their broader context, for example the need for treatment [51]. According to the DSM-IV-TR [12], the GAF value is useful in planning treatment, measuring the impact of treatment, and predicting outcome, but there is limited information available on the adequacy of GAF in prediction of outcome [19]. Information concerning the choice of level of care for different ratings could be given, for example a patient with a score of 1-30 is a potential candidate for inpatient care, a patient with a score of 31-69 a potential candidate for outpatient care, and a patient with a score of 70 and higher may be functioning too well to be a candidate for any treatment.

#### Gap in knowledge

Introductions to guidelines have been given little attention in international literature. Ground rules for rating have been little analysed and there is little information about how they are understood by different raters. It is not known what the result would be if international consensus panels of experts worked with ground rules.

### (3) Starting scoring at the top, middle or bottom level of the scale

It is known from methodology studies of questionnaire design that the ordering of response categories is a problem. Studies show a tendency to choose the both first listed response category ('primacy' effect) and the last listed response option ('recency' effect). Primacy effects are more likely in self-completion surveys [58]. A similarity in methodology problems exists for GAF and questionnaires [13]. Clinicians perform the rating by asking questions, and the GAF's deciles (with anchor points) are used as response categories. There is no common international norm for where to start; existing guidelines for GAF: (a) recommend starting at the top level of the scale with evaluation of whether the patient is worse than indicated by each of the decile's anchor points [12]; or (b) recommend starting at the bottom level [51]; or (c) give no instructions for where to start [5].

It may be hypothesised that starting from the top results in higher values than starting from the bottom and it is known that with questionnaires even seemingly minor changes can have a major impact [59]. An alternative approach would be to start in the middle of the scale (GAF = 50) and ask if the severity is worse or the patient is more healthy and then keep moving down or up the scale until the range that best matches the individual's symptom severity or level of functioning is reached. To double check, a look at the next upper or lower range would be taken.

#### Gap in knowledge

Information concerning the effects of starting the rating process at top, middle or bottom level is difficult to find.

### (4) Scoring for different time periods and of different values

#### Which time period?

In psychiatry, symptoms can change over time, for example over 24 h [16]. According to the DSM-IV-TR manual [12], the GAF score (in most instances) should be the level at the time of evaluation. The current level of functioning can be operationalised to the lowest level of functioning for the last week [12,38,50,51], which may be used to represent a baseline before onset of treatment [60]. It has also been suggested that symptom scales for the degree of severity of current illness should cover the past 3 days [61], but in acute care departments, even shorter time periods can be relevant [51].

The score for the last week may conflict with the patient's previous mental health, and fluctuations in the patient's condition may need to be scored several times over a longer period of time [62]. If this is not done, clinically useful information might be lost [63]. Scoring can also be done for time periods, for example for the last week and the past year [23]; this may cause considerable differences in scores [61] and so, when relevant, scoring can be done for more than one time period [23]. Examples of proposed time periods are: last year, last 6 months, at least a few months during the past year, and the preceding month [12,21,29,42,51].

Knowledge of the course of different conditions over time is essential [64]; for some patients and studies, scoring for longer periods may be appropriate. Longitudinal descriptions of the psychopathology can add information. The importance of premorbid level of functioning has been little explored and is rarely documented [3], but for chronic conditions, it is logical to consider adding scores for longer periods [65]. Depression can be scored by, for example: depression in the past year for 2 weeks or more, for much of the time in the past year, or for most of the days over a 2-year period [65]. For bipolar disorder, scoring of current symptoms is not enough and it is necessary to check for a past history of mania [66]. If psychosis has lasted for a longer period, the GAF score should be lower than the score given at admission for a first-time psychosis. For personality disorders, the stability of personality is a defining feature and a longitudinal perspective is essential in diagnosing [67]: scoring can be done for the past several years, the past 5 years, the 2 years before the interview, or the 'usual self' [67].

When the effect of treatment is being studied, GAF should be scored both before and after treatment [12]; scoring periods of between 3 and 12 months after discharge are suggested [65]. For patients under treatment for a longer period, scoring can be done every 2 or 3 months [63]. For example, outpatients who have not been given a GAF score in the last 90 days should be given a new score [42,68].

#### Gap in knowledge

The longitudinal dimension of using different GAF scores for different disorders has been little explored and existing guidelines give little instruction. There is little research data available about the time period that should be used for GAF rating or the criteria for choosing a specific time period. It is not known whether scoring should be done for the same time period for the GAF-S and GAF-F scales, whether scoring should be done for different time periods for the higher and lower ends of each GAF scale, or whether scoring should be done for different time periods for different anchor points.

#### Which value (lowest, highest or average)?

The aim of scoring should be to give a true image of the patient's mental health that will be useful for clinicians and research. As the severity of illness can vary over time, the question of which GAF value to record becomes relevant. Simple alternatives are the lowest, highest or average GAF for a time period. According to scoring instructions for GAF, when the current level of functioning is scored, the lowest score for the last week should be used; the lowest level of functioning is chosen because of its clinical relevance [51]. Rating GAF may mean choosing the lowest score for other specified time periods, for example the lowest level in the past month or for the worst week during the month prior to interview [3,37,39,63,69].

However, assigning the lowest GAF score is not without problems. It may give a wrong impression of both the overall mental situation and the present status [42]; the highest level of functioning should not be disregarded [12,31,39,57,70] as it may predict outcome [71]. For example, the highest level of functioning for at least a few months during the last year may be very predictive of outcome [19,52] and indicate the potential level of functioning [60]. Also, it has been reported that the highest level of functioning during the past year can be highly correlated with current level [19].

If the patient is not well described by either the highest or the lowest GAF for the last week, a solution may be to use more scores; for example, scores such as highest and lowest for the last year, the highest and lowest the patient has ever had, or scores for when the patient is symptomatic and asymptomatic. Rating of average functioning has also been proposed [29,50], for example, the average level of functioning during the previous 3 weeks [5,57]. If such scores describe the patient well, they can be added.

Internationally, both the single-scale and dual-scale GAF are in use. For the single-scale GAF, according to the manual for DSM-IV-TR [12] only one value should be recorded, namely, 'whichever is the worse' of the symptom and functioning values [5,12,21,22]. It is assumed that the GAF-S and GAF-F are comparable scales [16,27], so recording only the most severe of the GAF-S and GAF-F scores is in accordance with the general principle of using the most severe condition as the overall score [16]; however, the difference between the two scales is disregarded so it is not clear which factor of symptoms and functioning is being measured [52]. An alternative could be to record the average of symptoms and functioning levels [72], but this raises the question of whether or not symptoms and functioning have equal weight, and the importance of any weighting effect [73]. Although the values on each scale may be close [29], symptoms and functioning are different aspects of patient condition and they do not necessarily vary together [23], so in some countries a dual-scale GAF is used where both GAF-S and GAF-F are recorded [13].

In the clinical setting, comments can be added to a GAF score on why a particular score was chosen, which may be important when others take over treatment. It may also have an educational effect, add meaning to the scores, and improve inter-rater reliability [42]. However, it would be helpful if guidelines included a norm for the choice of score with more detailed information about which score to record; this is not an easy task, as mental illness is a multifaceted and complex problem. Deciding the criteria for such a norm is problematic.

#### Gap in knowledge

It is difficult to find empirical research aimed at finding the right GAF value (lowest, highest, or average), or combination of GAF values, to record for different applications. The potential applications for GAF scoring are wide ranging and include different diagnostic categories, the chronic and acutely ill, treatment decisions, prediction or measurement of outcome, choice of level of care, and measurement of case mix. Little is known about which score gives the best inter-rater reliability and validity, and it is not known whether separate GAF-S and GAF-F, or the lower of the two scores is best for treatment decisions and measurement of outcome, or how much weight should be given to GAF-S versus GAF-F for such applications.

### (5) The finer grading of the scale

The DSM-IV-TR, Veterans Administration and Norwegian guidelines have instructions for scoring within 10-point intervals, but instructions are limited [5,12,13,51]. Scoring within the 10-point intervals is open to subjective judgment and finer distinctions readily become somewhat random. In practice, clinicians tend to score around the decile or mid-decile divisions of the scale [42]. Patients who are scored in the same 10-point interval should be relatively homogenous in functioning, but functioning is a construct with many facets and when information for a more accurate score is lacking, intermediate scores in the deciles are chosen [63,74].

It is possible that more detailed verbal instructions would result in more accurate scores. An alternative to having more anchor points is to use categorical scales for scoring within the 10-point intervals, in which case the anchor points (with key words and examples of symptoms and functioning items) should be graded [13,75]. Both symptoms and functioning can be graded in different ways [76]. A categorical scale requires a decision about the number of categories; such scales often have five categories, for example: very marked, marked, neither marked nor weak, weak, or very weak. Numbers of categories other than five can also be considered [61,77]. More experienced raters may be able to make finer distinctions and score correctly with more categories, but scoring in the clinic is often carried out by people with different educational backgrounds [15,16,19-21,29]. An alternative procedure for scoring within 10-point intervals is found in the 'modified GAF' [24], which uses the number of criteria met: for example, for the interval 41-50, when one criterion is met the score should be 48-50 and when two criteria are met it should be 44-47.

#### Gap in knowledge

In the history of GAF, systematic work to improve scoring within 10-point intervals is limited and it is not known how to best score within 10-point intervals. This also applies to the use of categorical scales for scoring, which requires considerations concerning the nature and number of categories.

### (6) Different guidelines for different conditions

There can be a vast difference between the mental states of different patients. However, a dual-scale GAF scoring uses two straight lines (that is, a multidimensional phenomenon is scored in a two-dimensional way), which may not reflect this complexity. The answer to the problem is not necessarily to have more scales covering different aspects of, for example functioning, as this would require a more complex scoring process [13]. However, if guidelines for rating are not good enough, the value of an assessment instrument is reduced. It does seem appropriate to consider development of guidelines for different conditions.

Panels of experts aided by empirical data could develop norms with ranges of relevant GAF values. The comprehensibility of anchor points (with key words and examples) for different diagnostic group should be considered and it would be helpful to include examples of patients scored and not scored in each decile [13,77]. The reliability of scores is not necessarily the same for all diagnostic groups. To ensure assignment of the correct GAF value, advice could be given on how to obtain good information for each patient (for example which psychiatric interview to use). For some diagnostic groups, this can mean collecting more information than for others. Guidelines should have information on how to take different comorbid conditions into consideration.

If different GAF values are expected for different ages and sexes, this should be noted in the guidelines, but there is little information available about this. Different norms of functioning can represent different baselines against which the patient is evaluated, so, for example, instruments should be adapted to assessing older patients, to include scoring of dementia and happiness at the end of life [9]. Guidelines could also be different for different situations, for example for admission to inpatient departments and for community studies [13].

GAF should score impairment due to mental condition, but the effect of somatic and mental impairment can be interrelated and it can be difficult to distinguish between them [14]. The GAF rating should not be influenced by considerations on prognosis, previous diagnosis, presumed nature of the underlying disorder, or whether or not the patient is receiving medication or some other form of help [5,12,50,51].

#### Gap in knowledge

There is limited empirical information concerning the suitability of existing guidelines for different conditions, different groups of patients and patients with several other characteristics. The effect of adapting guidelines to these variations is not known. Having different guidelines for symptoms and functioning has been little explored.

### (7) Different languages and cultures

GAF has been translated into many languages, but languages encode meaning in different ways. Instruments should be adapted to different cultures and languages [6,7,40,73,78].

People from different cultures can answer in different ways when questions are asked, for a number of reasons [73,79], and this can have consequences for GAF values. It is important to understand illness explanations and help-seeking behaviours [80] within the patients' cultural framework and patients should be evaluated against what is 'normal' in their own culture. Cultural factors can be important for attitudes to disorder [81-83], and the use of GAF in multiethnic societies presents challenges to assessment [9].

Language differences may also present problems; a patient may be clearly psychotic when interviewed in their own language, but not when interviewed in a foreign language [83]. When translated into other languages, the guidelines for rating GAF, interviews for rating GAF, and GAF itself (for example anchor points with key words and examples) can be influenced. Translation of assessment instruments can involve translation, back translation, review and modification and guidelines are available for translating tests and assessment instruments [9,84].

#### Gap in knowledge

Little is known about the importance of translation and culture for GAF guidelines. The safety of international comparisons should be questioned. Meta-analyses based on data from countries with different languages and cultures may be influenced by these differences.

### Further development for GAF

We are a long way from having a comprehensive set of heuristic guidelines that could support the assessor in executing the scoring process [85], but progress in the study of the assessment process is anticipated [9]. Guidelines should be based on both theory, and empirical knowledge [85] about how each guideline works in practice. Development of new guidelines for GAF would be facilitated by first reviewing the literature about guidelines for psychological assessment, and extracting relevant points [6,7]. New empirical research could then be performed, for example by performing qualitative studies of the actual process of scoring, to search for items that are relevant for guidelines, while bearing in mind that if the scoring process is made too complex, errors are more likely to be introduced [76]. The existence of international guidelines would provide support to the implementation and use of the guidelines in different countries. Guidelines should reflect consensus on practice [7] and a draft of new guidelines for GAF should therefore be circulated widely to provide ample opportunity for comments [56]. A GAF scale with new guidelines should also be tested out for reliability and validity for different diagnoses, with different scorers, across different sites and with different patient populations. To study the effects of varying guidelines, knowledge of 'true' values would be useful and mean scores from expert panels can work as reference norms [29].

When designing a norm for the scoring process, it is important to consider which process can best achieve the aims. It is essential to first define the purpose of a scoring system. For example, a system that is mainly intended for clinical use should be viewed by clinicians as sensible and easy to use. However, having a short version of the guidelines for the clinic and more detailed guidelines for research could result in scores that are not directly comparable; evidence-based treatment is, by definition, based on research and this could pose a problem for its implementation.

A manual with more information about GAF and scoring of GAF could also be developed alongside the guidelines [86]. The requirement for guidelines to be short and concise makes it necessary to decide which information should be given in the guidelines and which in the manual. The manual can serve as principal source of information and might contain information about issues relating to GAF, such as history of its development; the theoretical basis; the comprehensiveness of GAF for different conditions; the reliability and validity of GAF with explanations for problems; statistical information for different diagnostic groups (mean value, standard deviation, range and statistical distribution, whether normal or skewed, and in which direction); information about which methods to use together with GAF (multimethod assessment is common); GAF values compared to values from other methods; implications of different GAF scores for treatment, with examples and thresholds of severity values defining when treatment is desirable; management use of GAF (for example in planning and comparison of case mix) [87]; rating by teams and individuals; use of GAF for patients with different cultural and linguistic backgrounds; and training material with descriptions of several cases with assigned GAF values.

Computerisation of assessment may well be the future. Assigning scores could begin with a visible GAF scale on the screen, where placing the cursor at different places along the scale reveals different windows with information about the criteria for scoring; clicking the mouse in one of these windows could make even more detailed information available in another window. The use of electronic patient records represents a possibility for new quality assurance methods. Some diagnoses are not combinable with high GAF scores; if such a diagnosis has been given, a warning could pop up on the screen if a GAF score that is too high is given. If a low GAF-S is given, a warning could pop up if a high GAF-F is given. A reminder may come up if the psychiatric record is completed for a new patient without having entered a GAF score. When a GAF score has not been given for an outpatient for the last 3 months, a reminder could pop up on the screen. Computer-based scoring of GAF can give high correlation with scoring based on clinical impression [88], but difficulties with computer-assisted assessment suggest a number of guidelines for users [41]. The International Test Commission has developed guidelines on computer-based and internet-delivered testing [89-94], but these guidelines were not developed with GAF in mind.

Work with a scoring instrument is not complete without testing or pilot study [82,95]. Alterations to the scoring process are not necessarily always improvements, and a pilot study is needed to reveal any additional changes that are necessary.

## Discussion

### Methods

Literature reviews can play a role in development of guidelines [96]. The present study can be defined as a systematic review [48,49]. Several important criteria for review articles are satisfied, such as defining the problem, informing the reader of the status of current research, identifying gaps and suggesting the next step [97].

An encompassing hand search of literature was done because it was considered that some relevant publications were likely not to be included in computerised databases. A combination of searching reference lists and reading publications has been considered the most thorough way of hand searching [98]. PubMed includes more than 500 psychology-related journals [99], but as the search showed few publications to deal specifically with guidelines for rating GAF, the search was continued in other databases. The citation tracking in Google Scholar is not completely reliable when it comes to listing the most frequently cited first, but screening of the first 1,000 results represents a thorough Google Scholar search. The search in PsycINFO added little new knowledge. The search in The Campbell Collaboration Library of Systematic Reviews added no new studies. The searches in PubMed, Google Scholar, The Campbell Collaboration Library of Systematic Reviews, and PsycINFO are reproducible. The search in PubMed, Google Scholar, and PsycINFO revealed that most of the publications were already identified by the thorough hand search (step (c) in Methods). In step (i), a stage was reached where new perspectives could not be identified by reading more publications; the situation is described by the term 'saturation' from qualitative research. It is not considered likely that publications that could have changed the results were missed as a result of the search process. The design and conduct of the present study protected against bias [47,48].

### Better guidelines for GAF

The literature review identified the state of knowledge for GAF guidelines and a review of this type can be valuable in work to develop better guidelines. In the history of GAF, limited focus has been given to development of guidelines and currently available guidelines are short. In the clinic, the primary goal of the assessment process is to contribute to the solution of a person's problems [100]. A generic and global scoring system, such as GAF, that covers the range from positive mental health to severe psychopathology has advantages for clinical practice (for example, routine quality assessment of treatment, supplementing scales that give more detail) [75], research (for example, comparison of treatment outcome across diagnoses), and policy and management planning (for example, allocation of resources, measurement of case mix in psychiatric organisations). For GAF to have such a broad range of applications, it must be good enough for the purpose. It is important not to simply dismiss GAF because of problems concerning either the instrument itself [13] or guidelines; existing scales can be dismissed too lightly [72].

A scoring system must be robust enough to allow for scorer bias and more random errors of measurement. If GAF is not good enough, a given change in GAF value would not necessarily reflect a corresponding change in severity. Subjectivity in scoring should be kept to a minimum; some scorers can be unwilling to give a low score because of the negative labelling of clients [22] and clinicians who do most of their work with one patient category may use their experience as a norm. Improved consistency of scoring can be achieved locally by delivering courses in rating GAF [22], but the risk of variation between different local standards will remain. Improved guidelines have the potential to reduce such bias.

The aim of better guidelines is to make scores more reliable, to improve comparability of scores (for example across organisations and from different studies), to make combination of scores in meta-analysis safer, help in assigning more accurate scores (choosing better between individual points in the 10-point ranges), to provide more accurate information for the choice of intervention and evaluation of treatment results, and to be of help in the education and training of assessors. However, it is not a matter of course that new guidelines will give much better GAF scores.

The clinical situation is not just about having a perfect scoring system; it is equally important to earn the respect and trust of the patient [70]. New guidelines should not be destructive for the clinician-patient relationship. They should also be adaptable and tolerate changes in clinical practices; information for scoring should be easy to obtain; and the scoring process should not be too time consuming. Evidence-based medicine has shown that examples of successful implementation of guidelines exist, but also that implementation is not always successful [101]. It is important that once new guidelines for GAF have been developed, they are implemented effectively.

### Factors other than the process of scoring

The present review has focused on guidelines for rating GAF, but other factors can also play a part in the choice of GAF value. Factors that have not been treated include: (1) characteristics of the patient interview and the importance of collecting information from different sources; (2) characteristics of the rater, i.e. professional background, training and motivation, groups, or individuals score; and (3) properties of GAF (discussed in a previous study) [7,13,19,20,23,34,36,39,57,58,61,77,102-105].

## Conclusions

The guidelines that are currently available for rating GAF are not the result of a sophisticated development, but guidelines are important for reliable assessments. There are few published studies dealing specifically with guidelines for rating GAF. This study presents a number of points that are relevant for new guidelines and show a significant potential for development.

International panels of experts have a role to play, and a manual for GAF can be developed. Computerisation of the scoring process can offer advantages for rating. In light of the current situation, care should be exercised when comparing outcomes across facilities and also with international comparison, and meta-analyses. More work is needed to develop improved guidelines for rating GAF.

## Competing interests

The author declares that he has no competing interests.
